# The mechanism of solid acid-catalyzed bamboo sawdust liquefaction under polyol systems

**DOI:** 10.3389/fbioe.2024.1372155

**Published:** 2024-03-20

**Authors:** Bin Wu, Hongwei Tang, Yijia Huang, Mengke Zhao, Long Liang, Zhanghong Xie, Linshan Wei, Guigan Fang, Ting Wu

**Affiliations:** ^1^ Institute of Chemical Industry of Forest Products, Chinese Academy of Forestry, Nanjing, China; ^2^ Nanjing Forestry University, Nanjing, China; ^3^ Sichuan Academy of Forestry, Chengdu, China; ^4^ Yibin Paper Industry Co., Ltd., Sichuan Province Engineering Technology Research Center of Bamboo Pulping and Papermaking, Yibin, China; ^5^ China Pulp and Paper Research Institute Co., Ltd., Beijing, China; ^6^ Shandong Huatai Paper Co., Ltd., Dongying, Shandong Province, China

**Keywords:** solid acid catalysts, bamboo sawdust, biomass liquefaction, polyol systems, crystalline conversion

## Abstract

Solid acid catalysts are widely used in the field of biomass catalytic conversion owing to their advantages of low environmental pollution, easy separation and reusability. Nevertheless, there are relatively few studies on the mechanism of solid acid liquefaction for biomass. In this study, the effect of acid strength and acid amount of various solid acids on the liquefaction efficiency has been investigated using waste bamboo sawdust generated from the pulp and paper industry as the raw material. In addition, the physicochemical changes of cellulose, hemicellulose and lignin during the reaction process of bamboo sawdust have been studied, and the liquefaction mechanism of bamboo sawdust under the action of various solid acids has been concluded. As a result, the liquefaction efficiency of bamboo sawdust under the polyol system of PEG400/propanetriol is mainly related to the acid strength of the solid acid, and the greater the acid strength of the solid acid, the better the catalytic effect on the bamboo sawdust, in which the residual amount of bamboo sawdust liquefaction catalyzed by the SPA catalyst is only 17.72%. Noteworthy, the most difficult component to liquefy is the crystallization of natural cellulose I into cellulose II during the reaction process, which is the primary obstacle to the complete liquefaction of bamboo sawdust by solid acid. Overall, these findings are valuable for the high value utilization of waste bamboo sawdust in the pulp and paper industry, as well as the application of solid acid catalytic technology for biomass.

## 1 Introduction

Biomass liquefaction is an effective method of utilizing biomass and is the basis for the preparation of various bio-based materials. In this process, acid is usually added as a catalyst, since it can effectively reduce the reaction temperature and improve the liquefaction efficiency of biomass (Pan, 2011). The commonly used homogeneous acids are sulfuric acid, hydrochloric acid, and phosphoric acid (Jensen et al., 2010; Kumar et al., 2017; Zhang et al., 2019), whose drawbacks are the difficulty of catalyst recycling, the amounts of wastewater in the production process and the corrosion of the equipment, while the solid acid catalysts have the advantages of low environmental pollution, easy separation. In addition, although inorganic acids are cheaper than solid acids, the higher costs of environmental protection equipment, production and maintenance do not provide a clear economic advantage in terms of the overall process.

Solid acid-catalyzed liquefaction of biomass has been extensively reported. For example, Santander et al. showed that solid phosphoric acid supported by mesoporous silica catalyzed the liquefication of cellulose, with levogluconone detected in more than 85% of the liquid product (Santander et al., 2019). In addition, the hydrothermal pyrolysis of SO_4_^2-^/Fe_2_O_3_, SO_4_^2-^/Al_2_O_3_, SO_4_^2-^/TiO_2_, SO_4_^2-^/ZrO_2_ and SO_4_^2-^/SnO_2_ was studied by Yang et al. (2015), with the highest acidic strength, SO_4_^2-^/SnO_2_, yielding 11.0% and 26.8 %for HMF and glucose, respectively. Li et al. (2020) reported a multifunctional catalyst (Cl-MCMB-SO_3_H) for the hydrolysis of cellulose. Compared with the catalyst MCMB-SO_3_H, the Cl^−^ in the catalyst Cl^−^MCMB-SO_3_H favored the formation of hydrogen bonds, which reduced the activation energy of cellulose hydrolysis by about 20 kJ/mol, improving the hydrolysis efficiency with a high reducing sugar yield of 70.3%. Additionally, Liu et al. (2011) presented a multifunctional solid acid catalyst (Ru/Cs_3_PW_12_O_40_) to catalyze cellulose after ball milling, with sorbitol yields up to 43%, which showed good catalytic performance even after 5 times of reuse. From the current research, the solid acids used for biomass catalysis are mainly concentrated in the similar class of solid acids. In particular, there is a lack of systematic research on the compositional relationship between different types of solid acids for biomass liquefaction, and the structure of solid acids and biomass liquefaction, which leads to the selection and preparation of solid acids for biomass liquefaction mainly relying on experience.

The utilization of bamboo for pulp and paper production is a promising development in Asia, especially in China. It is expected that in 5 years, bamboo-based pulp and paper products will reach 10 million tonnes per year. Accordingly, a million tonnes of bamboo sawdust will be generated, which cannot be utilized at high value. The aim of this study was to investigate the effects of acid strength and acid quantity of various solid acid types on the liquefaction efficiency of bamboo sawdust. In addition, the physicochemical changes of cellulose, hemicellulose and lignin during the reaction process of bamboo sawdust were also taken as the focus. Finally, the liquefaction mechanism of bamboo sawdust under various solid acid conditions was proposed.

## 2 Experimental section

### 2.1 Material

*Bambusa emeiensis* were provided by YIBIN PAPER INDUSTRY CO., LTD. (Sichuan, China). Moreover, the bamboo sawdust (BS) was derived from *Bambusa emeiensis* after being crushed in a pulverizer and sieved 20–80 mesh (0.18mm–0.85 mm). The composition of BS as follows 39.62% glucan, 13.69% xylan and 31.16% lignin.

### 2.2 Catalysts preparation

ZrO_2_, SnO_2_, TiO_2_ and Fe_2_O_3_ were obtained by sintering Zr(OH)_4_, Ti(OH)_4_, Fe(OH)_3_ and Sn(OH)_4_ in air, respectively. ZrO_2_, SnO_2_, TiO_2_ and Fe_2_O_3_ were impregnated with 1 mol L^-1^ sulfuric acid for 1 h (metal oxide: sulfuric acid = 1 g: 15 mL), and then calcined in a muffle furnace. Typically, the calcination temperature was 500 °C for Fe, Sn, Ti and 600°C for Zr, as well as the calcination time was 3 h for Zr, Fe and 4 h for Ti, but 5 h for Sn. The final prepared superacid catalysts were named as SO_4_^2−^/Fe_2_O_3_, SO_4_^2−^/TiO_2_, SO_4_^2−^/ZrO_2_ and SO_4_^2−^/SnO_2_, respectively.

5.0 g of diatomaceous earth (Chengdu Kolon Chemicals Ltd.) and 28.8 g of 85% phosphoric acid (Chengdu Kolon Chemicals Ltd.) was mixed and stirred to form a viscous paste mixture, which was then calcined in a muffle furnace at 300 °C for 48 h to yield white solid. Afterwards, the resulting product was cooled to room temperature in dry environment and ground into powder to produce solid phosphoric acid “SPA”.

Phosphotungstic acid (PTA) was purchased in Chengdu Kolon Chemicals Ltd (Sichuan, China).

All catalysts have been passed through a sieve with 0.15 mm aperture.

### 2.3 Solid acid-catalyzed liquefaction of bamboo

The heterogeneous catalytic degradation reaction system was formed in an oil bath at 170°C for 18 h. 5 g bamboo sawdust was added to a mixture of 30 g of PEG 400 and 10 g of glycerol, and 1.5 g solid acid catalyst was added. In addition, the filtrate was filtered and the residue was washed with 1,4-dioxane/water (4:1, v/v) until the reacted detergent was colorless. After that, the detergent was recovered by rotary evaporation at 60°C and reused. The filter residue was dried at 105°C for 8 h and then calcined at 550°C before calculating the liquefaction conversion. The mass burned off is the mass of organic matter in the bamboo sawdust residue, and this calculation ignores the effect of solid acid loss.

$$R\left(\%\right)=\frac{\left(m_{r}-m_{c}\right)}{m_{0}}*100\%$$ (1)

Where the R represents the residue yield (%), m_r_ refers to the residue mass (g), m_c_ stands for the residue mass after burning (g) and m_0_ is the bamboo sawdust mass (g).

### 2.4 Analysis method

#### 2.4.1 NH_3_-TDP analysis

The acid strength and acid content of the catalysts were determined by ammonia temperature-programmed desorption (NH_3_-TPD) over a temperature range of 100°C–700°C. And the measuring instrument is an AutoChem II 2920 from Mike Company.

#### 2.4.2 Chemical component analysis

Concentrations of dextran, xylan, and lignin in solid samples and post-reaction residues were assessed using standard technique from the Renewable Energy Laboratory (NREL). Sugar content was assessed using an Agilent high performance liquid chromatography (2160II, CA, United States) and a Bio-rad Aminex PHX-87H sugar column, with signal detection using an oscillometric refractive index detector. Acid-soluble lignin was identified using an UV spectrophotometer (UV-Vis, T6 series, Beijing spectrum analysis General Instrument Co.).

#### 2.4.3 FT-IR analysis

The IR spectra of the samples were measured in the wavenumber range of 400–4,000 cm^-1^ (Nicolet iS10, Thermo Fisher Scientific, Waltham, MA, United States). Solid samples were prepared as 1 wt% KBr pressed tablets, and IR spectra of liquid samples were determined by removing the solvent (PEG 400/glycerol = 3/1) background.

#### 2.4.4 XRD analysis

X-ray diffraction (XRD) patterns of the samples were obtained on a Bruker D8 Focus X-ray diffractometer utilizing Cu K radiation. In detail, the samples were scanned in 4° increments with a counting time of 1 min per spot over a range from 10° to 80°.

#### 2.4.5 Thermogravimetric analysis

Samples were collected on a TA SDT650 thermogravimetric analyzer. The samples were ramped from room temperature to 900°C in N_2_ atmosphere (40 mL/min) at a rate of 10°C/min.

## 3 Results and discussion

### 3.1 Solid acid and liquefaction rate

**FIGURE 1:**
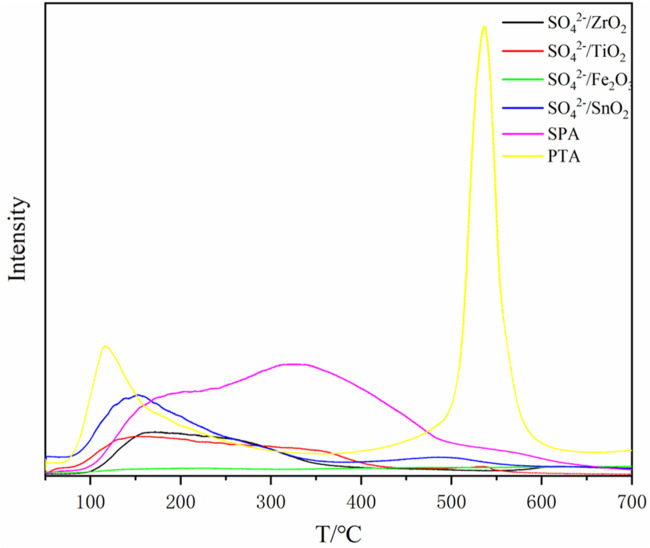
NH_3_-TPD curves of catalysts samples.

The liquefaction effects of different solid acids on the same mass (1.5 g) of bamboo sawdust are shown in Table 1. Different kinds of solid acids show great differences on bamboo sawdust, among which the residue rates of loaded acid SPA, heteropolyacid PTA and SO_4_^2-^/M_x_O_y_ are 17.72%, 36.7% and 56.96%–68.02%, respectively. Therefore, among these catalysts, SPA exhibits the greatest effect on the liquefaction of bamboo sawdust. However, from the NH_3_-TPD profiles (Figure 1), it can be seen that the heteropolyacid PTA is the most acidic as it has obvious NH_3_ resolution peaks in the high temperature region relative to SPA and SO_4_^2-^/M_x_O_y_. According to previous studies, the stronger the acidity, the better the effect on biomass liquefaction (Lin et al., 2022). Nevertheless, for PTA and SPA, two typical proton solid acids, the opposite result is observed. The “free P_2_O_5_” on the surface of SPA readily interacts with low molecular organic matter. As a result, SPA is susceptible to forming complexes with low molecular organic matter, which makes it easier to come into contact with the substrate. In addition, water is produced during the reaction, which hydrolyzes some of the solid acid on the surface of the SPA to produce liquid phosphoric acid (Santander et al., 2019). Then, the liquid phosphoric acid comes into contact with the reactants and catalyzes the liquefaction of bamboo sawdust. As a result, the residue rate is the lowest under SPA catalyzation. These results indicate that the loaded solid acid has obvious advantage in catalyzing the liquefaction of bamboo sawdust in polyol alcohol system.

**TABLE 1:** Influence of catalysts on liquefaction effect.

Catalysts	Residue yield/%
SPA	17.72
PTA	36.72
SO_4_^2-^/SnO_2_	56.96
SO_4_^2-^/ZrO_2_	61.59
SO_4_^2-^/Fe_2_O_3_	65.21
SO_4_^2-^/TiO_2_	68.02
blank	73.99

The desorption temperature is related to the acid strength of the solid acid. Generally, a higher desorption temperature indicates a more difficult desorption of NH_3_ and therefore a higher acid strength of the solid acid (Liu et al., 2014). In the low-temperature region of 100°C–200°C, all types of solid acids show resolved peaks, which are mainly due to the physical adsorption of NH_3_ on the surface of solid acid as well as its chemisorption on the weakly acidic sites. In the middle and high temperature region, compared with other solid acids, PTA solid acid has a strong absorption peak in the high temperature region around 550°C, and thus has the highest acid strength. In addition, the NH_3_ resolution peak of SPA is higher than that of SO_4_^2-^/SnO_2_ in the middle and high temperature region. Therefore, the order of acid strength of these three solid acids is PTA > SPA > SO_4_^2-^/SnO_2_.

In Table 1, the residue rates of bamboo sawdust after liquefaction of the three types of solid acids were ranked as follows: SPA (17.72%) > PTA (36.72%) > SO_4_^2-^/SnO_2_ (56.96%). The acidity of PTA is higher than that of SPA, but in contrast, the residual rate after PTA treatment is higher. This is probably because SPA is a loading acid that produces small molecules of water during the liquefaction of bamboo sawdust, which hydrolyzes part of the solid acid on the surface of SPA to produce liquid phosphoric acid (Santander et al., 2019). In addition, the liquid phosphoric acid comes into contact with the reactants and catalyzes the liquefaction of bamboo sawdust under their joint action with the lowest residual rate. However, the residual rate of bamboo sawdust liquefaction catalyzed by PTA is smaller than that of catalyzed by SO_4_^2-^/SnO_2_, which may be attributed to the fact that PTA is more acidic than SO_4_^2-^/SnO_2_.

As shown in the NH_3_-TPD profiles (Figure 1), compared with SO_4_^2-^/ZrO_2_, SO_4_^2-^/Fe_2_O_3_ and SO_4_^2-^/TiO_2_, the SO_4_^2-^/SnO_2_ solid acid has a resolved peak in the high-temperature region at 500°C, and thus the acid strength is the largest. Moreover, SO_4_^2-^/ZrO_2_ and SO_4_^2-^/TiO_2_ have obvious resolved peaks in the middle and low temperature regions, with similar peak areas. In contrast, SO_4_^2-^/Fe_2_O_3_ has no distinct resolved peak in the whole NH_3_-TPD curve. Therefore, the acid intensity of SO_4_^2-^/M_x_O_y_ solid acid was ranked as follows: SO_4_^2-^/SnO_2_ > SO_4_^2-^/ZrO_2_, SO_4_^2-^/TiO_2_ > SO_4_^2-^/Fe_2_O_3_.

From Table 1, the liquefaction residue rate of SO_4_^2-^/SnO_2_ solid acid was 56.96%, while the residue rates of SO_4_^2-^/ZrO_2_, SO_4_^2-^/TiO_2_ and SO_4_^2-^/Fe_2_O_3_ were 61.59%, 68.02% and 65.21%, respectively. These results indicated that the stronger the acidic site of solid acid, the better the liquefaction effect of bamboo sawdust in polyol system. In addition, the liquefaction residue rate of SO_4_^2-^/Fe_2_O_3_ was essentially the same as that of SO_4_^2-^/ZrO_2_ and SO_4_^2-^/TiO_2_, suggesting that the weak acid sites of the solid acid had no impact on the catalytic effect of bamboo sawdust in the polyol system.

In summary, the liquefaction efficiency of bamboo sawdust under the polyol system of PEG400/glycerol is mainly related to the acid strength of the solid acid and the type of the solid acid with less correlation to the total amount of the acid in the solid acid: (1) the stronger the solid acid, the better the catalytic effect; and (2) the loading of solid phosphoric acid, the better the catalytic effect. This is because water molecules will be generated during the reaction process, and the water molecules will lead to the hydrolysis of solid phosphoric acid to generate liquid phosphoric acid, which will increase the reaction rate and make the bamboo sawdust liquefied more fully under the polyol system.

### 3.2 Analysis of chemical composition of residues

**FIGURE 2:**
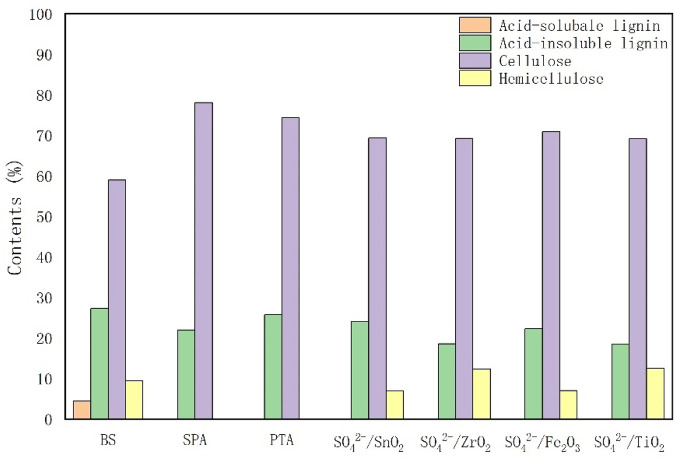
Effects of different catalysts on the mass fractions of chemical composition in liquefied residues.

In order to investigate the effect of different solid acids on the liquefaction process of bamboo sawdust, the biomass fractions in the solid acid-catalyzed residue were determined. As shown in Figure 2, the relative content of cellulose in the residue increased in all catalyst groups compared with that of bamboo sawdust, with the most significant increases in the SPA and PTA groups. These results indicate that cellulose is the most difficult part to liquefy during the liquefaction of bamboo sawdust. Acid-soluble lignin was fully reacted in all catalyst groups, while the content of acid-insoluble lignin varied. In addition, the hemicellulose content varies among the catalyst groups, with complete reaction of hemicellulose in the SPA and PTA groups, and changes in the hemicellulose content of the other groups compared to the bamboo sawdust. In order to quantify the effect of solid acid types on the content of three major components in the liquefaction of bamboo sawdust, the relative contents of these components in bamboo sawdust (before liquefaction) and their residues (after liquefaction) were quantified (Table 2). The relative content of the three major elements (X) in the residue (after liquefaction) as a percentage of the pre-liquefaction bamboo sawdust was calculated according to the following equation (Niu et al., 2011):

$$X=M_{1}\times R\times 100\%$$ (2)

where M_1_ represents the relative content of the three major elements in the residue and R refers to the yield of the liquefied residue.

**TABLE 2:** Chemical composition of bamboo sawdust and liquefaction residues.

Sample	Cellulose/%	Hemicellulose/%	Lignin/%	
			Acid soluble lignin/%	Acid insoluble lignin/%
BS	58.86	9.41	4.43	27.30
SO_4_^2-^/TiO_2_	47.23	8.51	0	10.75
SO_4_^2-^/Fe_2_O_3_	46.18	4.53	0	12.05
SO_4_^2-^/ZrO_2_	42.61	6.77	0	9.74
SO_4_^2-^/SnO_2_	39.51	3.77	0	10.51
PTA	27.29	0	0	8.16
SPA	13.82	0	0	4.25

*The chemical composition were calculated according to Eq. 2.

Cellulose is most difficult to liquefy because of its crystalline structure, which makes it difficult for the liquefying agent to act on it. In Table 2, the cellulose content in bamboo sawdust was 58.86%, which decreased under all conditions by adding solid acid. The cellulose contents of bamboo sawdust decreased to 39.51%–47.23% catalyzed by solid acid SO_4_^2-^/M_x_O_y_. In comparison, the cellulose content in bamboo sawdust was 27.29% using PTA with higher acid strength as catalyst. Therefore, the increase in solid acid strength facilitated the liquefaction of cellulose in bamboo sawdust. When SPA was used as a catalyst, the cellulose content was only 13.82%, which was attributed to the hydrolysis of solid phosphoric acid on the surface of SPA to small molecule phosphoric acid, promoting the hydrolysis of cellulose (Espinosa et al., 2013). Comparative analysis of the data show that cellulose in bamboo sawdust is the most difficult to be liquefied under the polyol system of PEG400/glycerol, which is the main obstacle restricting the complete liquefaction of bamboo sawdust by solid acids.

The hemicellulose content of bamboo sawdust is 9.41%, which is relatively low. Moreover, the addition of various solid acid catalysts can promote the liquefaction of hemicellulose in bamboo sawdust. Notably, there is no hemicellulose in the residues after solid acid catalysis by PTA and SPA. Therefore, the hemicellulose in bamboo sawdust can be completely liquefied by solid acid catalysis with sufficient acidity in the polyol system.

The lignin in bamboo sawdust could be categorized into acid-soluble lignin and acid-insoluble lignin, with contents of 4.43% and 27.30%, respectively. The addition of various solid acids completely liquified the acid-soluble lignin in bamboo sawdust, indicating that the acid-soluble lignin in bamboo sawdust was more easily liquefied. Moreover, the solid acid catalysts could also promote the liquefaction of acid-insoluble lignin in bamboo sawdust. Among them, the liquefaction effect of SPA was the best, which could reduce the acid-insoluble lignin content in bamboo sawdust from 27.30% to 4.25%. The lignin content in bamboo sawdust was extremely low. Studies have shown that lignin is more easily liquefied by acid and this incomplete liquefaction may be due to the long liquefaction reaction time. Additionally, the decomposed small-molecule lignin was further condensed to large-molecule compounds (Xie et al., 2014), which remain in the residue. Therefore, acid-insoluble lignin is present in the residue.

Conclusively, the addition of solid acids promoted the liquefaction of the three main elements in bamboo in the polyol system of PEG400/glycerol. Acid-soluble lignin was the easiest to liquefy, and the addition of all types of solid acids resulted in its complete liquefaction. This was followed by hemicellulose and acid-insoluble lignin, which could be completely liquefied by solid acids with increasing acidic strength.

Cellulose is the most difficult to be liquefied, which may be due to the fact that cellulose molecules are aggregated in the form of micro protofibrils, in which the crystalline and amorphous regions alternate, and the amorphous portion of cellulose is easily hydrolyzed during acid hydrolysis, leaving the crystalline fraction behind (Li, 2004; Kuthi and Badri, 2014). Meanwhile, during the liquefaction process, the liquefaction products undergo condensation reactions with lignin, condensation products tend to remain on the crystalline cellulose in the residue (Xie et al., 2014). These factors make it difficult for the residual cellulose to come into contact with the liquefiers for further reaction.

### 3.3 FT-IR analysis of liquefaction residues

**FIGURE 3:**
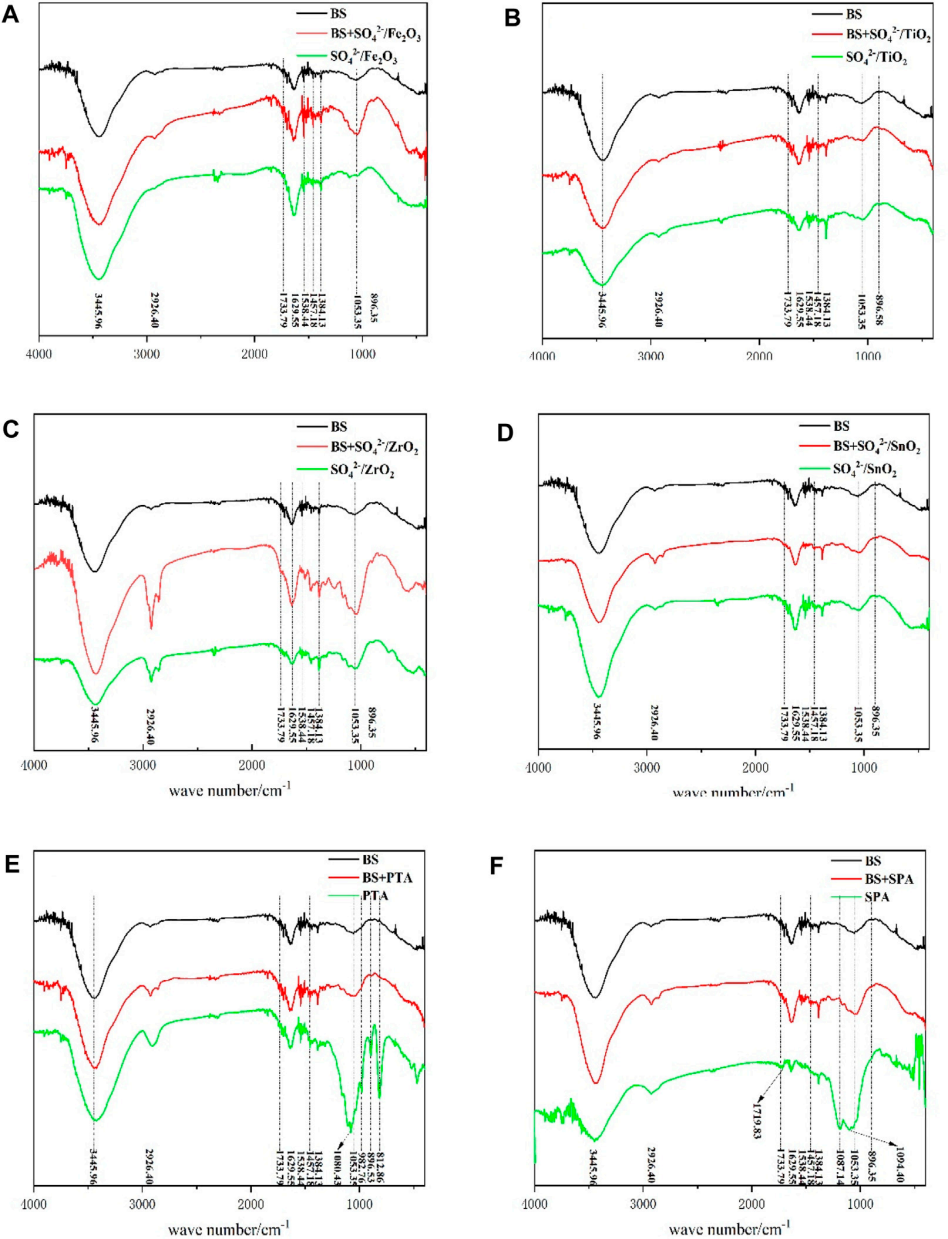
Infrared spectra of BS, BS and catalyst mixture as well as liquefaction residue.

In order to compare the changes of functional groups on the surface of residues after liquefaction with different catalysts, the liquefaction residues, bamboo sawdust and the mixture of bamboo sawdust and solid acid with 1.5 g of catalyst were characterized by IR spectroscopy, and the results were shown in Figure 3.

The infrared absorption peaks of the main components of BS (cellulose, hemicellulose and lignin) can be seen in the infrared spectra. The broad peaks between 3,300 and 3,500 cm-1 belong to the stretching vibration peak of -OH. Moreover, the absorption peak at 2,926 cm-1 is attributed to the vibration absorption peak of -CH3, while the absorption peak appearing at 1733 cm-1 should be attributed to the uncoupled C=O stretching vibration, which is also the absorption peak of characteristic functional groups of hemicellulose (Wen et al., 2011; Xie et al., 2014). Notably, the peak intensity is not high due to the small content in BS. The absorption peaks at 1,629 cm-1, 1,518 cm-1 and 1,457 cm-1 belong to the benzene ring skeleton vibration, corresponding to the lignin structure in bamboo sawdust (Zhang et al., 2010). Absorption peak at 1,384 cm-1 is attributed to C-H bending vibration, and the peak at 1,053 cm-1 belongs to C-O stretching vibration, which is also a characteristic absorption peak of cellulose and hemicellulose in the infrared (Latif et al., 2014). One of the peaks at 896 cm-1 belongs to β-glucoside bond vibration, which is also the infrared characteristic absorption peak of cellulose (Chen et al., 2011). Meanwhile, the mixture of BS and solid acid catalyst were characterized by IR spectroscopy. It was found that the addition of solid acid catalyst had no effect on the position and intensity of the IR characteristic absorption peak of BS (cellulose, hemicellulose and lignin). Therefore, the effect of solid acid-catalyzed liquefaction of BS could be evaluated by comparing the changes in the IR characteristic absorption peak of BS and liquefaction residue.

As shown in Figure 3, the IR spectral curves of pure BS, the mixture of BS and catalyst, and the residue of solid acid liquefaction are shown from top to bottom, respectively. Solid acids such as SO42-/ZrO2, SO42-/TiO2, SO42-/Fe2O3 and SO42-/SnO2 can catalyze the liquefaction of bamboo sawdust, but the residue rate is high. The liquefaction rate was low, and only part of cellulose, hemicellulose and lignin were liquefied (Figure 2; Table 2). As a result, the position of IR characteristic peaks was basically unchanged and the intensity did not change much, which was consistent with the detection results of residue compositions.

When PTA with high acidity was used as catalyst, the IR absorption peak of hemicellulose at 1733 cm-1 basically disappeared compared with the BS spectrum, indicating that hemicellulose had been completely reacted. In addition, the absorption peaks of cellulose and hemicellulose at 1,053 cm-1 also disappeared, while new absorption peaks, i.e., the IR absorption peaks of cellulose, appeared at 1,080 cm-1, 1,053 cm-1 and 896 cm-1. Meanwhile, a new IR absorption peak was observed at 812 cm-1, which should be attributed to α-glycosidic bond vibrations (Wen et al., 2011), indicating an increase in cellulose content in the residue. This result is consistent with the detection of the residue compositions.

When SPA with better liquefaction effect was used as catalyst, the IR absorption peak at 1733 cm-1 of hemicellulose was found to be completely disappeared when compared with the BS IR spectrum, indicating that hemicellulose was completely liquefied. At the same time, a new peak reappeared at 1719 cm-1, which was attributed to the absorption peak of conjugated C=O in aromatic nuclei. After liquefaction, the surface lignin was enriched on the residue surface by the products of polycondensation. In addition, the IR characteristic absorption peaks of cellulose and hemicellulose at 1,053 cm-1 of bamboo sawdust catalyzed by SPA disappeared, and two new peaks at 1,187 cm-1 and 1,094 cm-1 were formed with high peak intensity. These two peaks are the infrared characteristic absorption peaks of cellulose (Wen et al., 2011), indicating that the content of cellulose in liquefaction residue is increased. On the other hand, the intensities of the IR characteristic peaks of 1,629 cm-1, 1,518 cm-1 and 1,457 cm-1 in the liquefied residue were reduced compared with those in the BS, indicating lignin degradation, which was consistent with the results of residue components.

Above infrared analysis results showed that with the increase of the acidity of the solid acid, the hemicellulose and lignin in bamboo material disappeared or weakened, while the characteristic absorption peak of residual cellulose was enhanced and the liquefaction effect was improved. All these indicated that the stronger the acid strength of solid acid, the better the liquefaction effect of bamboo sawdust under the polyol system of PEG400/glycerol.

### 3.4 XRD analysis of liquefaction residue

**FIGURE 4:**
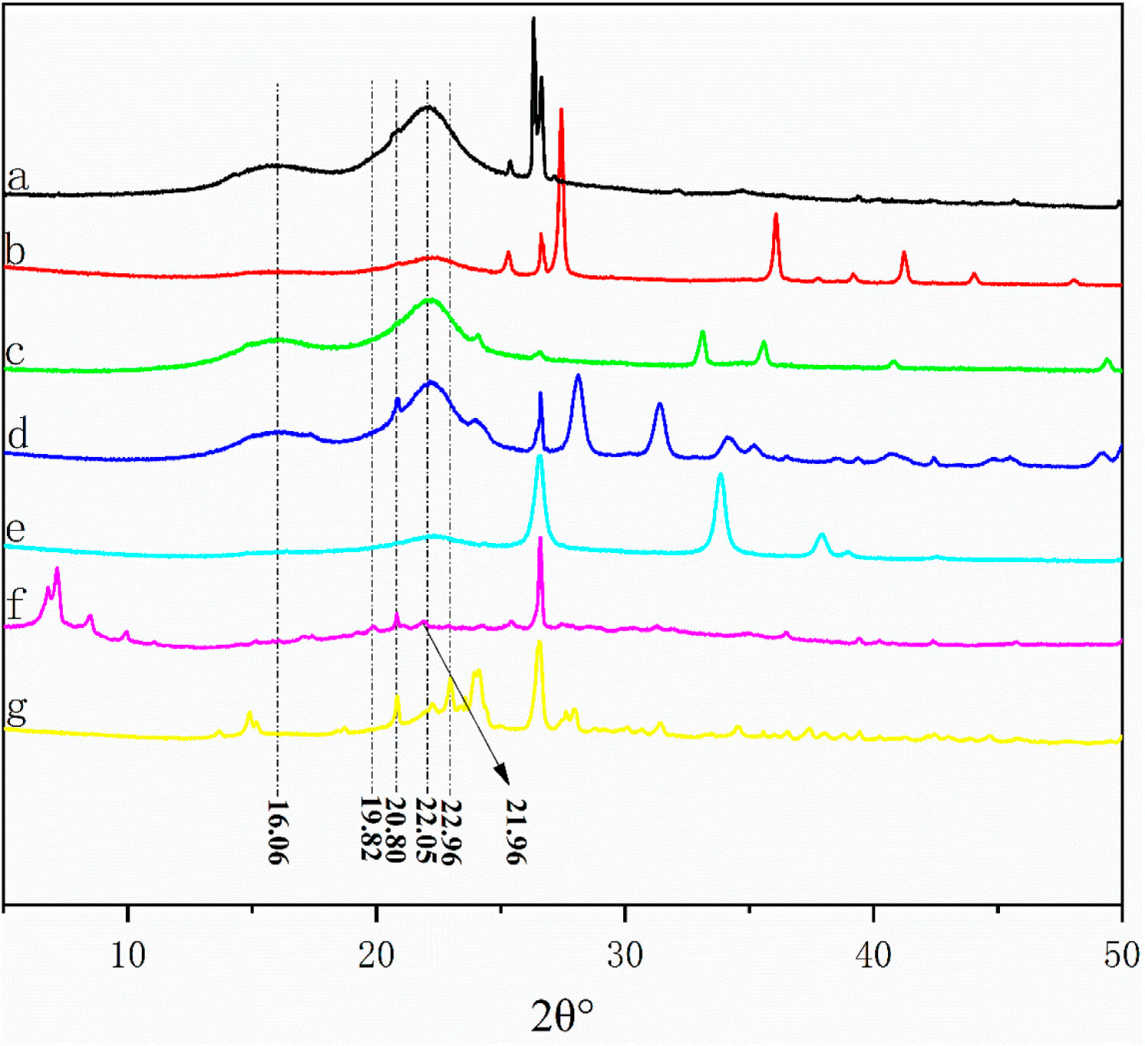
XRD patterns of BS and liquefied residues: **(A)** BS; **(B)** residue from SO_4_^2-^/TiO_2_ system; **(C)** residue from SO_4_^2-^/Fe_2_O_3_ system; **(D)** residue from SO_4_^2-^/ZrO_2_ system; **(E)** residue from SO_4_^2-^/SnO_2_ system; **(F)** residue from PTA system; **(G)** residue from SPA system.

Figure 4 shows the diffraction intensity curves of the liquefaction residue and BS under the condition of 1.5 g catalyst dosage. According to the XRD pattern of BS, the main diffraction angles 2θ° are 16.06°, 22.05° and 26.30°, among which 16.06° and 22.05° are the diffraction peaks of natural cellulose Ⅰ (Langan et al., 2001; Nishiyama et al., 2002). In addition, all samples have a narrow diffraction peak at 26.30°, which may be the absorption peak corresponding to the BS containing SiO2 (Tymchyshyn and Xu, 2010).

Compared with BS, the liquefaction residue of SO42-/ZrO2, SO42-/TiO2, SO42-/Fe2O3 has diffraction peaks of cellulose Ⅰ at 22.05° and 16.06°. It is noteworthy that the shape of the peak remains almost unchanged, but there is a decrease in strength, which may be due to the liquefaction of some cellulose in the bamboo. In addition, this may also be due to the presence of a large amount of solid acid catalyst in the residue.

With the increase of liquefaction efficiency, the diffraction absorption peak belonging to cellulose Ⅰ is disappeared at 16.06° when SO42-/SnO2 is used as catalyst, indicating that part of cellulose Ⅰ also reacts.

When PTA is used as a catalyst, the characteristic diffraction peaks of natural cellulose at 22.05° disappear, while the diffraction peaks of cellulose II at 21.96° and 20.80° appear (Langan et al., 2001), which may be attributed to the transfer of the metastable structure of natural crystalline cellulose Ⅰ from bamboo sawdust to thermodynamical stable cellulose Ⅱ (Isogai et al., 1989; Langan et al., 2001).

A similar phenomenon is observed in the SPA liquefaction residue when the residue rate is only 17.72%. The new diffraction absorption peaks are appeared at 21.96°, 20.80° and 22.96°. Meanwhile, the new characteristic diffraction peaks belonging to cellulose are sharp, indicating that the crystalline cellulose on the surface of the residue has been reacted, so the crystallinity of the remaining cellulose has been improved, which explains the difficulty of cellulose liquefaction in bamboo sawdust.

From the above XRD analysis results, it can be inferred that with the increase of solid acid acidity, the amorphous part of cellulose will be liquefied firstly during the catalytic process, leaving the crystalline part of natural cellulose, due to the alternation of crystalline and amorphous regions of natural cellulose. Then, when the acidity of the solid acid is further enhanced, part of the natural crystalline cellulose will be liquefied. Part of it is converted into the thermodynamically more stable cellulose II, which is the reason why bamboo sawdust is difficult to be completely liquefied.

### 3.5 Thermogravimetric analysis of liquefaction residue

**FIGURE 5:**
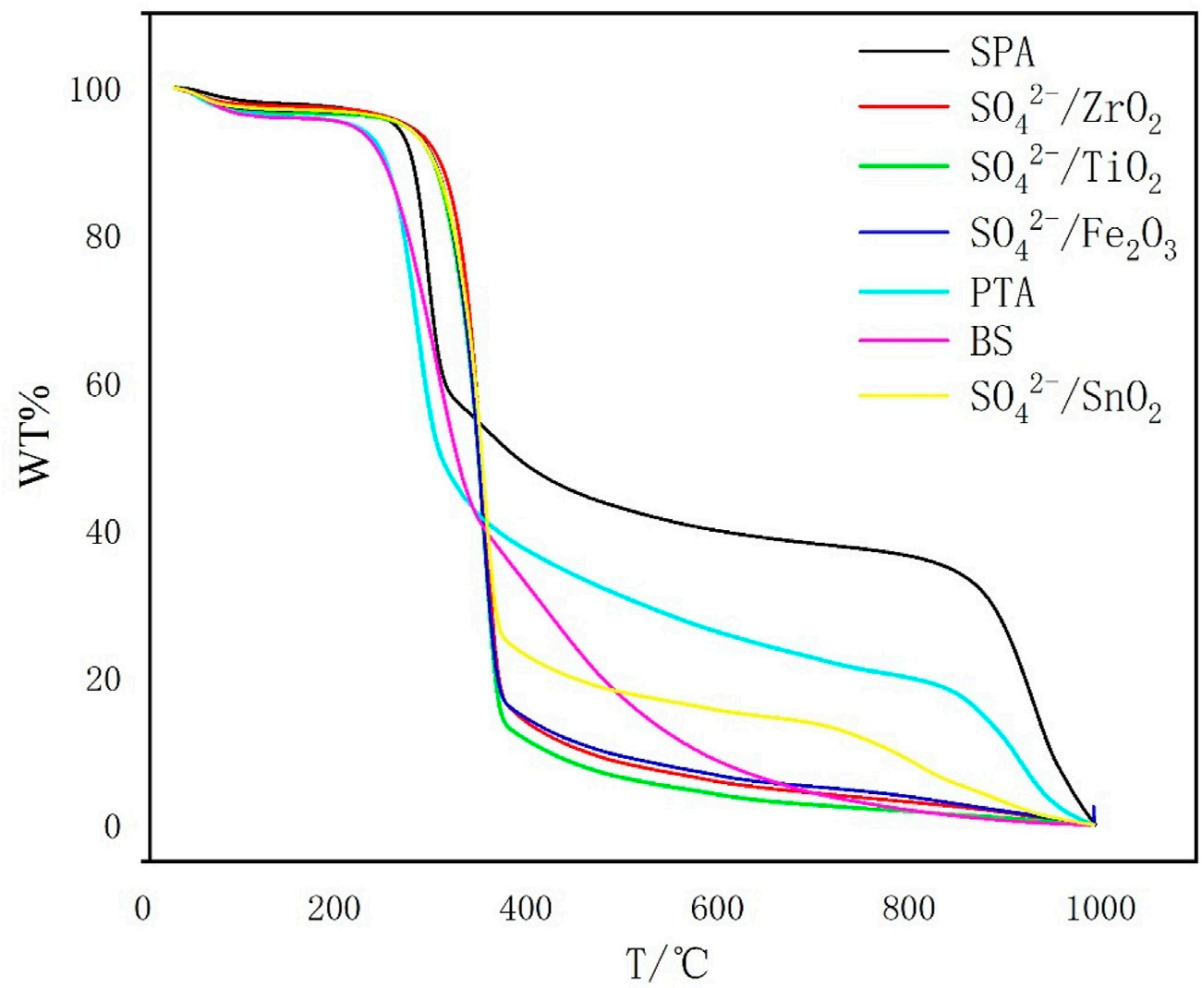
TG curves of bamboo sawdust and solid acid liquefaction residues.

Figure 5 shows the TG curve of liquefaction residue and the BS to remove the inorganic salts as well as the remaining solid acid from the residue at a catalyst dosage of 1.5 g. Based on the data in Figure 3, the weight loss of all the samples can be calculated and the results are shown in Table 3. From Figure 5, it can be seen that the weight loss zone of bamboo sawdust can be divided into three parts. In stage a, the weight loss rate is 4.85%, which is ascribed to the evaporation of water from the residue. In stage b, the weight loss rate is as high as 90.81%, which is mainly due to the pyrolysis of lignin, cellulose and hemicellulose according to the compositional analysis in Figure 2. In stage c, the weight loss rate is only 4.34%, which is primarily ascribed to the pyrolysis of charcoal residue after bamboo pyrolysis.

**TABLE 3:** Weight loss rates for all samples at different temperature ranges.

Samples	Temperature/°C			
	Weight loss ratio/%			
	Stage a	Stage b	Stage c	Stage d
BS	33–210	210–700	700–1,000	/
	4.85	90.81	4.34	/
SO_4_^2-^/TiO_2_	33–210	210–380	380–1,000	/
	3.67	82.87	13.46	/
SO_4_^2-^/Fe_2_O_3_	33–210	210–380	380–1,000	/
	3.39	80.23	16.38	/
SO_4_^2-^/ZrO_2_	33–210	210–380	380–1,000	/
	2.93	80.66	16.41	/
SO_4_^2-^/SnO_2_	33–210	210–380	380–700	700–1,000
	3.34	71.79	11.12	13.75
PTA	33–210	210–355	355–830	830–1,000
	4.74	53.70	22.64	18.92
SPA	33–210	210–315	315–820	820–1,000
	2.87	37.04	24.25	35.84

The weight loss of SO_4_^2-^/MxOy solid acid, SPA and PTA liquefaction residue is basically the same as that of the bamboo sawdust at stage a in the weight loss zone, which is mainly due to the evaporation of water from the residue, which is mainly due to the evaporation of water from the residue.

Compared with the weight loss of bamboo sawdust in the b-zone stage, the significant weight loss temperature range of all solid acid residues in this stage is shortened, with significantly faster weight loss and lower weight loss rate (Figure 3), which is inversely correlated with the acid strength. This is attributed to the liquefaction of cellulose, hemicellulose and lignin in bamboo cuttings catalyzed by solid acid. Notably, the greater the acid strength, the more complete the liquefaction, and the more complete the destruction of the main structure of bamboo sawdust. Therefore, in stage b, the temperature interval of weight loss is significantly shortened, and the rate of weight loss is significantly accelerated, while the weight loss rate is reduced.

For stage c, the TG curves of all catalyst liquefaction residues and bamboo cuttings are relatively flat, and the weight loss rate is relatively slow, which might be due to the pyrolysis of the more thermally stable organic matter in the liquefaction residues.

As for stage d, it can be seen from Figure 5 that only the liquefaction residue of SO42-/SnO2, SPA and PTA have thermogravimetric regions in the high-temperature region at this stage. The relationship is as follows: the range of weight-loss temperature, weight loss rate and weight loss rate of the residue in stage d increase with the decrease of the liquefaction residue rate, which indicates that the three elements in the liquefied bamboo sawdust produce products that are difficult to be pyrolyzed with the increase of the acidity of the solid acid. By analyzing the weight loss curve in stage d, it is found that the weight loss rate of SPA liquefaction residue is 35.84%. From Figure 2, it can be seen that the composition of SPA liquefaction residue is 21.96% acid-insoluble lignin and 78.04% cellulose. Therefore, it can be judged that the material of heat weight loss at this stage is mainly cellulose. Meanwhile, XRD results show that the cellulose in the residue is mainly composed of cellulose Ⅱ with relatively high crystallinity. Previous studies have shown that cellulose is less prone to weight loss during low-temperature pyrolysis when the crystallinity of cellulose is high (Mukarakate et al., 2016) cellulose is less prone to weight loss during low-temperature pyrolysis when the crystallinity of cellulose is high. This explains well why the liquefied residues of SO42-/SnO2, PTA and SPA increase by an interval of weight loss at 700 °C.

## 4 Conclusion

In conclusion, the effects of different solid acids as catalysts on the liquefaction rate of bamboo biomass have been investigated. Consequently, the liquefaction efficiency of bamboo sawdust was mainly related to the acid strength of the solid acid under the polyol system of PEG400/glycerol. Notably, the higher the acid strength of solid acid, the better the catalytic effect on bamboo sawdust. After the liquefaction of bamboo sawdust, the residual amount of SPA catalyst was only 17.72%, which catalyzed the liquefaction of the majority of the biomass in bamboo sawdust. Additionally, the most difficult component to liquefy was the crystalline conversion of natural cellulose I to cellulose II during the reaction process, which was the main obstacle limiting the complete liquefaction of bamboo sawdust by solid acid.

## Data Availability

The raw data supporting the conclusion of this article will be made available by the authors, without undue reservation.

## References

[B1] Chen W. Yu H. Liu Y. (2011). Preparation of millimeter-long cellulose I nanofibers with diameters of 30-80 nm from bamboo fibers. Carbohyd Polym. 86 (2), 453–461. 10.1016/j.carbpol.2011.04.061

[B2] Espinosa S. Kuhnt T. Foster E. Weder C. (2013). Isolation of thermally stable cellulose nanocrystals by phosphoric acid hydrolysis. Biomacromolecules 14 (4), 1223–1230. 10.1021/bm400219u 23458473

[B3] Isogai A. Usuda M. Kato T. Uryu T. Atalla R. H. (1989). Solid-state CP/MAS carbon-13 NMR study of cellulose polymorphs. Macromolecules 22 (7), 3168–3172. 10.1021/ma00197a045

[B4] Jensen J. Morinelly J. Aglan A. Mix A. Shonnard D. R. (2010). Kinetic characterization of biomass dilute sulfuric acid hydrolysis: mixtures of hardwoods, softwood, and switchgrass. Aiche J. 54 (6), 1637–1645. 10.1002/aic.11467

[B5] Kumar S. Ahluwalia V. Kundu P. Sangwan R. S. Kansal S. K. Runge T. M. (2017). Improved levulinic acid production from agri-residue biomass in biphasic solvent system through synergistic catalytic effect of acid and products. Bioresour. Technol. 251, 143–150. 10.1016/j.biortech.2017.12.033 29274853

[B6] Kuthi F. Badri K. (2014). Effect of cooking temperature on the crystallinity of acid hydrolysed-oil palm cellulose. AIP Conf. Proc. 1614, 456–462. 10.1063/1.4895240

[B7] Langan P. Nishiyama Y. Chanzy H. (2001). X-Ray structure of mercerized cellulose II at 1 Å resolution. Biomacromolecules 2, 410–416. 10.1021/bm005612q 11749200

[B8] Latif S. Nahar S. Hasan M. (2015). Fabrication and electrical characterization of bamboo fiber-reinforced polypropylene composite. J. Reinf. Plast. Comp. 34 (3), 187–195. 10.1177/0731684414565941

[B9] Li H. Zhang X. Wang Q. Yang D. Cao Q. Jin L. (2020). Study on the hydrolysis of cellulose with the regenerable and recyclable multifunctional solid acid as a catalyst and its catalytic hydrolytic kinetics. Cellulose 27 (1), 285–300. 10.1007/s10570-019-02777-3

[B10] Li X. (2004). Physical, chemical, and mechanical properties of bamboo and its utilization potential for fiberboard manufacturing. Thesis. Baton Rouge, LA: Louisiana State University. 10.31390/gradschool_theses.866

[B11] Lin T. Meng F. Zhang M. Liu Q. (2022). Effects of different low temperature pretreatments on properties of corn stover biochar for precursors of sulfonated solid acid catalysts. Bioresour. Technol. 357, 127342. 10.1016/j.biortech.2022.127342 35605770

[B12] Liu M. Deng W. Zhang Q. Wang Y. Wang Y. (2011). Polyoxometalate-supported ruthenium nanoparticles as bifunctional heterogeneous catalysts for the conversions of cellobiose and cellulose into sorbitol under mild conditions. Chem. Commun. 47 (34), 9717–9719. 10.1039/c1cc12506k 21789299

[B13] Liu X. Lang W. Long L. Hu C. L. Chu L. F. Guo Y. J. (2014). Improved catalytic performance in propane dehydrogenation of PtSn/γ-Al_2_O_3_ catalysts by doping indium. Chem. Eng. J. 247, 183–192. 10.1016/j.cej.2014.02.084

[B14] Mukarakate C. Mittal A. Ciesielski P. Budhi S. Thompson L. Iisa K. (2016). Influence of crystal allomorph and crystallinity on the products and behavior of cellulose during fast pyrolysis. ACS Sustain. Chem. Eng. 4, 4662–4674. 10.1021/acssuschemeng.6b00812

[B15] Nishiyama Y. Langan P. Chanzy H. (2002). Crystal structure and hydrogen-bonding system in cellulose iβ from synchrotron X-ray and neutron fiber diffraction. J. Am. Chem. Soc. 124, 9074–9082. 10.1021/ja0257319 12149011

[B16] Niu M. Zhao G. Alma M. (2011). Thermogravimetric studies on condensed wood residues in polyhydric alcohols liquefaction. Bioresources 6 (1), 615–630. 10.15376/biores.6.1.615-630

[B17] Pan H. (2011). Synthesis of polymers from organic solvent liquefied biomass: a review. Renew. Sust. Energ Rev. 15 (7), 3454–3463. 10.1016/j.rser.2011.05.002

[B18] Santander A. Alvarez M. Gutierrez V. Volpe M. A. (2019). Solid phosphoric acid catalysts based on mesoporous silica for levoglucosenone production via cellulose fast pyrolysis. J. Chem. Technol. Biot. 94 (2), 484–493. 10.1002/jctb.5795

[B19] Tymchyshyn M. Xu C. (2010). Liquefaction of bio-mass in hot-compressed water for the production of phenolic compounds. Bioresour. Technol. 101 (7), 2483–2490. 10.1016/j.biortech.2009.11.091 20031393

[B20] Wen J. Xiao L. Sun Y. Sun S. N. Xu F. Sun R. C. (2011). Comparative study of alkali-soluble hemicelluloses isolated from bamboo (Bambusa rigida). Carbohydr. Res. 346 (1), 111–120. 10.1016/j.carres.2010.10.006 21109235

[B21] Xie J. Hse C. Shupe T. Qi J. Pan H. (2014). Liquefaction behaviors of bamboo residues in a glycerol-based solvent using microwave energy. J. Appl. Polym. Sci. 131, 40207. 10.1002/app.40207

[B22] Yang F. Li Y. Zhang Q. Sun X. Fan H. Xu N. (2015). Selective conversion of cotton cellulose to glucose and 5-hydroxymethyl furfural with SO_4_ ^2−^/M_x_O_y_ solid superacid catalyst. Carbohyd Polym. 131, 9–14. 10.1016/j.carbpol.2015.05.036 26256154

[B23] Zhang J. Deng H. Lin L. Sun Y. Pan C. Liu S. (2010). Isolation and characterization of wheat straw lignin with a formic acid process. Bioresour. Technol. 101 (7), 2311–2316. 10.1016/j.biortech.2009.11.037 20004567

[B24] Zhang Y. Liu Z. Liu H. Hui L. Wang H. (2019). Characterization of liquefied products from corn stalk and its biomass components by polyhydric alcohols with phosphoric acid. Carbohyd Polym. 215, 170–178. 10.1016/j.carbpol.2019.03.096 30981342

